# The Patient's Experience of the Psychosocial Process That Influences Identity following Stroke Rehabilitation: A Metaethnography

**DOI:** 10.1155/2014/349151

**Published:** 2014-01-28

**Authors:** E. Hole, B. Stubbs, C. Roskell, A. Soundy

**Affiliations:** ^1^Department of Physiotherapy, University of Birmingham, 52 Pritchatts Road, Edgbaston, Birmingham B15 2TT, UK; ^2^School of Health and Social Care, University of Greenwich, London SE10 9LS, UK

## Abstract

*Background and Purpose.* Patient experience is increasingly being recognised as a key health outcome due to its positive correlation with quality of life and treatment compliance. The aim of this study was to create a model of how patient's experiences of rehabilitation after stroke influence their outcome. *Methods.* A metaethnography of qualitative articles published since 2000 was undertaken. A systematic search of four databases using the keywords was competed. Original studies were included if at least 50% of their data from results was focused on stroke survivors experiences and if they reflected an overarching experience of stroke rehabilitation. Relevant papers were appraised for quality using the COREQ tool. Pata analysis as undertaken using traditional processes of extracting, interpreting, translating, and synthesizing the included studies. *Results.* Thirteen studies were included. Two themes (1) evolution of identity and (2) psychosocial constructs that influence experience were identified. A model of recovery was generated. *Conclusion.* The synthesis model conceptualizes how the recovery of stroke survivors' sense of identity changes during rehabilitation illustrating changes and evolution over time. Positive experiences are shaped by key psychosocial concepts such as hope, social support, and rely on good self-efficacy which is influenced by both clinical staff and external support.

## 1. Introduction

Each year around 110,000 people in England are affected by a stroke [1], meaning stroke affects between 176–216/100000 of the United Kingdom (UK) population [2] and around 80,000 are admitted to a hospital [3]. Approximately a third of individuals who experience a stroke will die within 3 months [4]. In the UK, stroke is the largest single cause of disability [5] with an annual cost to society of approximately *£*8.9 billion, around half of this cost representing direct care of the patient [6]. Experiencing a stroke and its aftermath can be devastating for patients and their families and may be associated with physical, social, and psychological consequences. Rehabilitation offers a chance for an individual to recover and/or adapt to their situation following a stroke [7]. It is essential that researchers understand the experience of stroke survivor's during rehabilitation and understand the patient's psychosocial needs. The value of qualitative research for this purpose and for informing policy and practice within healthcare is increasingly being recognised [8–11]. A particular value of synthesising qualitative data is found in examining the patient experiences of illness and their subjective beliefs [7, 8, 12, 13].

Patient experience identified through qualitative research has highlighted a need to focus on distinct areas of health care provision, including understanding respect, equality, access, and information to services, safety, choice, shared decision making, support, and representation [15–19]. Despite this call and focus there has been a lack of emphasis on the patient experience, a problem that has been recently acknowledged [1, 20]. The current emphasis on measuring important health related outcomes utilising standardised and predetermined questions (e.g., quality of life, satisfaction surveys) is often set to the health care professional's agenda. Such outcomes may not be valued by patients or carers [21–23] or worse it may prevent a health care professional listening to or valuing the individual's personal experience [24]. Further to this, at present there is no guidance as to how to take into account the personal experience of the individual [25, 26]. For instance the ICF system attempts to encompass the social functioning of the individual but does not focus on an individual's subjective experiences within healthcare [27]. For this reason there is increasing recognition that a better understanding of the patient's experiences is needed. This will help transform services helping them to achieve higher quality, in a safer environment and with more efficient processes [28–30].

A stroke can negatively influence an individual's sense of self, including one's ability to reflect upon the illness, make positive adjustments, taking control, and accepting any disabilities [31–33] and such an influence can last years after the stroke [34]. In addition to this, an individual's perception of their world can be changed challenging their relationships and social roles with others [32, 35]. For instance the stroke can remove their professional identity and other significant identities (e.g., athletic identities) by affecting the ability to engage in activities that previously were enjoyed [36].

The psychosocial components of rehabilitation, such as expectation, sense of identity, and acceptance are important psychological concepts which are often recognised as “unmet needs” [37–41]. These form a crucial part of a stroke survivor's experience. It is important that clinicians' are able to consider how the concept of identity and other psychological concepts can be used to inform the development of care and satisfaction for the patient. One way of achieving this would be through reviewing the existing literature in a way that focuses on and values the individual's experience.

In recent years a range of different methods for synthesising qualitative research have developed [42–44]. These different methods are evident in the literature [24, 40, 45] considering individuals who have experienced a stroke. However, this previous research has not considered how an individual's identity can be influenced by their interaction and integration with the social world or how psychosocial concepts may impact on this experience as the patient transitions through rehabilitation. A metaethnography represents one way to achieve this because the process has become an effective way of interpreting the findings of multiple studies in the search of new theories or concepts [13, 44, 46, 47].

*Aim.* The aim of this metaethnography review was to consider how a patient's experience of stroke rehabilitation influences and evolves their identity and to consider the different psychosocial concepts and interactions that may influence this.

## 2. Methods

We followed the traditional series of seven phases from conception of an idea through to the expression of the synthesis [57]. Within the paper we illustrate these phases within three stages [58]. These stages included (1) systematic search, (2) critical appraisal, and (3) synthesis. Each stage is detailed below.

### 2.1. Stage 1: Systematic Search

A multipronged approach was used as recommended by Campbell et al. [47]. The following electronic databases were searched from January 2000 until October 2012: AMED, CINAHL Plus, Medline (revised), EMBASE, ASSIA, IBSS, Biological Nursing Index, ProQuest Nursing & Allied Health Source, Social Sciences Abstracts, Sociological Abstracts, Science Citation Index, and Social sciences citation Index. The search terms can be observed in Table 1. Hand-searching of included article's reference lists was employed and where possible authors were contacted when a study could not be located.

**Figure 1:**
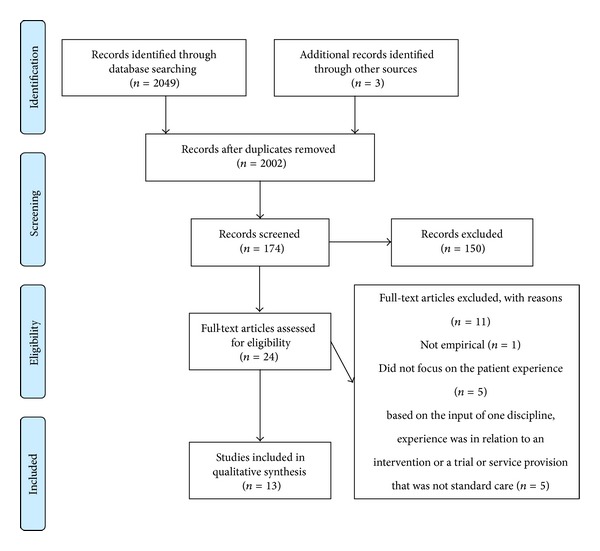
A PRISMA diagram for the study. This is based on PRISMA 2009 flow diagram and shows the search results across all four databases with numbers of articles retrieved and excluded at each stage of the selection process [14]. For more information, visit http://www.consort-statement.org/.

Figure 1 provides the combined database results within a Preferred Reporting Items for Systematic Reviews and Metaanalyses (PRISMA) flow diagram [14].

#### 2.1.1. Eligibility Criteria

Studies were included if they

- reported on patients' experience of rehabilitation after stroke following the year 2000. This date represents a major policy [59] shift towards patient-centred care within the NHS;
- utilised empirical qualitative data from primary research source such as interviews, focus groups, or ethnographic research;
- focussed on patient experiences reporting at least 50% of the result section with a focus on patients' experience and perceptions;
- considered the experience of rehabilitation provided by a number of health care professionals as part of an operating stroke service;
- published in a peer-reviewed journal in English.

#### 2.1.2. The Synthesis Papers

Thirteen studies met the inclusion criteria. The characteristics of the thirteen included studies are presented in Table 2. The location of the studies is shown and apart from Sabari et al. [48] carried out in the USA, the remainder were conducted within Western Europe. Five of the studies [38, 39, 49, 51, 55] used a single interview to collect their primary data, one of the studies [53] used a focus group, and seven of the studies [26, 37, 48, 50, 52, 54, 56] used repeated data collection over time. All studies undertook interviews, except one, which included video analysis from within a community stroke club setting [48].

Three studies [26, 37, 38] focused specifically on younger stroke survivors/patients (age range 37–65). Only two studies [60, 61] had a lower age limit of 60 years old. There were a number of topics used to guide the semistructured interviews and focus groups. Five studies [37, 39, 50–52] documented the patient's initial experiences of having the stroke, the event, and its impact.

### 2.2. Stage 2: Critical Appraisal and Quality Assessment

The Consolidated Criteria for Reporting Qualitative Studies [62] (COREQ) was used to assess the quality of all of the included studies. The COREQ was specifically developed to promote explicit and comprehensive interview/focus group reporting in qualitative research. It increases the reviewer's sensitivity to aspects of the research practice and identifies any flawed studies by looking for outliers in the data [10]. A scoring system was used for each item in each domain, each of the domains in the COREQ assessment tool was scored equally; for example, the details of the interviewer from domain one was given the same weighting as the theoretical framework of the study, domain two. Where an item was reported in the paper it was given a score of 1 but in the case of under reporting or where no report was given on that item the paper was given a score of 0. Each of the items in each domain was scored and a total reported for each paper out of a possible maximum score of 32.  Table 3 provides an overview of each study's methodological score.

No studies were fatally flawed [10] and all thirteen studies were included in the synthesis. Items were consistently reported as poor within domain one (research team and reflexivity) which included a description of the interviewer's relationship to the participant and the interviewer's characteristics. This finding was not considered sufficient to warrant further consideration within our analysis [10, 47, 58].

### 2.3. Stage 3: The Synthesis

During this phase 3 levels (1st, 2nd, and 3rd order) of interpretations were made [57, 58, 63]. The interpretations clarify the source of the information and encourage an in depth understanding of the original data in order to begin to form constructs [60, 63, 64]. The thirteen papers were examined in chronological order and two tables illustrating the first and second order interpretations were constructed keeping the findings separate (these findings are available from the primary author). In each table a separate column was used for notes. The supervising author (XX) assisted with the verification of interpretations made, recording techniques used, and development of third order interpretations. Key metaphors and concepts from the studies were juxtaposed to establish any relationship between the data [57]. To achieve this, the interpretation tables were used and the papers analysed chronologically to form an interpretation grid. Both the tables and grid are available from the primary author. The grid was filled analysing each paper in a separate row and each column represented a second order construct.

The included studies were amenable to reciprocal translation, as they all related to similar concepts and experiences [57]. Using the interpretation grid the concepts of the original studies were brought together, interpretations were made by the author as to where similarities in the data were, and key themes and subthemes were created. An attempt was made to retain the language used by the papers whilst creating new metaphors in the analysis [61]. Once translated the data was assessed for themes that followed a line of argument. A “line of argument synthesis” is defined as a new model, theory, or understanding from the synthesis of data using metaethnography [64]. This was developed by the supervising author utilising previous theoretical constructs, including the paradox of chronic illness [13] and the evolution of an individual's identity [46].

## 3. Findings

Two key themes were identified: the evolution of identity and the psychosocial constructs that influence experience. Each theme contains subthemes translated from the interpretation grid.  Table 4 that follows shows the thematic breakdown.

### 3.1. Theme 1: The Evolution of Identity

#### 3.1.1. Subtheme 1a: Sense of the Individual's Identity

A sense of the individual encompasses the whole person, their individual factors such as age and comorbidities and the activities and hobbies of their previous life. Participants suggested their broader human needs were not met and that they were not acknowledged as individuals in the rehabilitation experience [53, 54]. A more recent concept in some of the studies, the “individual recovery model,” is considered a direct response to this and an opportunity for professionals to understand how stroke survivors see a way to their recovery [55, 56]. The model suggests that an individual combines knowledge of themselves and their life experience with professional opinions and advice to create a path and understanding for their own recovery. Essentially this concept is not a new one; all the synthesised studies had the needs and wishes of the individual as key to successful and satisfying experience within rehabilitation. Indeed, many of the older studies also acknowledged that individuals had their own ideas of recovery and rehabilitation [37, 39, 48, 53]. These wishes are clearly associated with their hopes and expectations. For instance, a participant in the study by Bendz [37] states “*I want to get back to my usual self…the one I used to be.*” (Female, 64 years, after 3 months).

#### 3.1.2. Subtheme 1b: Being at the Dividing Line

This subtheme considers a key aspect of adjustment for individuals who have experienced a stroke, when they acknowledge the meaning of the stroke in their lives. This subtheme occurs in the patient reported experience at times of transition when self-realisation (acknowledgment and understanding of one's present situation) is heightened due to exposure to a new reality, such as going home after rehabilitation, when adjustments and new insights are required [52]. Transition, adjustment and the learning associated with these experiences appear to be significant for the individual in the rehabilitation process. By being able to problem solve and practice tasks, self-esteem and confidence are improved [56], as is self-efficacy. Expectations are high for individuals at the time of transition where the adjustments that follow require recognition of the “new” self. The role of the professional and the family seems crucial at this time (see Table 3) to assist the individual to rebuild and restructure their world through this experience [51].

#### 3.1.3. Subtheme 1c: Rebuilding, Restructuring, and Identity

Within this subtheme individuals acknowledge the situation that their stroke caused and consider the future and begin a process of adaptation and practice. Individuals with a stroke reported getting to a point following the acknowledgement of their situation, during or following rehabilitation where they consider the role and meaning of what their life will be. Individuals require time to adjust to this realisation [38, 49, 50, 54]. They utilise experiences [26, 51] to acquire this understanding and begin to attempt adaption to their stroke through practice. The desire for reintegration with aspects of their identity that can be restored is undertaken. This desire is an important motivating force. For instance, Erikson et al. [26] highlighted a participant at 12 months after stroke who stated “*Your goal is to live a normal life, just as it used to be. Your children should not need to think that it is an awkward old man that they are walking together with. You do not want to deviate more than before. It's important for oneself too*” (page, 836).

#### 3.1.4. Subtheme 1d: Reintegrating Identity with Their Life

Individuals' desire for reintegration to their social world after stroke was found to inspire engagement in rehabilitation and gave the feeling of increased energy and meaning in an individual's life [39, 50]. Individuals did not talk about their experience of participation but of belonging, having a sense of identity and an occupation [26, 38]. It appears that more than lacking in participation, stroke survivors described experiencing a lack of meaning to the tasks they practised in recreation and therapy [39, 55]. There was a great value to feeling a sense of belonging during the process of change that occurs with rehabilitation. The concept of belonging and acceptance of a “new” self appears important in the participant's experience of rehabilitation after a stroke. It is enhanced with occupation and success in activities of daily living by improving self-esteem and confidence [52].

### 3.2. Theme 2: Psychosocial Constructs That Influence Experience

#### 3.2.1. Subtheme 2a: Social Support

The partnerships and support experienced varied between studies and was provided by a network of people. The variability was probably due to the studies representing a cross-section of international data from acute rehabilitation, to, services for chronic stroke survivors. Support from professional staff and the individual's network of friends and family took on many forms. It included instrumental and emotional support as well as access to timely but potentially long term services providing information and resources. Frequently the participants highlighted the positive experience of having skilled staff available to provide feedback and encouragement [48, 52, 53, 55]. Further, the different ways that participants experienced support within their rehabilitation affected their ability to regain autonomy and reconcile their sense of self. Erikson et al., illustrate the vulnerability of a patient: “*I become very uncertain even though I know I'm right. My feeling is that the staff think that I understand less than I actually do*” (page 833).

#### 3.2.2. Subtheme 2b: Autonomy

In the majority of studies autonomy was highlighted as a key factor that influenced outcome by the majority (11/13) of studies. Individuals recognised having autonomy helped to build self-esteem and confidence instead of traditional paternalistic models of care constraining their independence and self-determination [49]. The ability to make their own choices and solve their own problems led to participants taking increased responsibility and improving their outcomes [52]. Participants identified that partnerships with professional staff and shared decision making improved their experience [54]. So through gaining autonomy participants were better able to reconcile their sense of “self” and this acted as a primary way to move on from the current self towards evolving the self.

#### 3.2.3. Subtheme 2c: Coping and Adjustment

Adjusting to and managing the effects of the stroke were expressed in several ways. Individuals were both able to look forward to the future and identify an ability to cope. Burton [50] suggests that “*when new coping mechanisms had been established and were perceived to be successful, informants felt more able to do things” *(page, 305). Other patients needed to express multiple losses. Patients described a loss of role, loss of belonging, loss of control, and the loss of ability described as the “paralysed everyday” [38, 39]. Patients described dependency on others and feelings of helplessness, further to this, the burden of being a burden (the negative thoughts and feelings associated with being a burden on someone else) and isolation [37, 51]. Indeed, six studies [26, 38, 50–52, 54] identified that patients did not want to be a burden on family members or friends. This perception clearly affected an individual's ability to cope. Although, the concept of “burden of burden” and its impact on well-being requires further investigation [26].

#### 3.2.4. Subtheme 2d: Hope

Hope, described by participants in respect of what they wished to happen and their expectations, involved a sense of moving forward continuously from dependence to independence similar to that described by coping [49]. The participant's expectations of training and the role that professionals had in their rehabilitation was high and observed specifically when describing their disappointment in the therapy input on going home or reduced opportunities for recreation [39, 55]. Many of the studies contained first order constructs of hope by the participants and the concept of hopelessness when finding themselves not progressing in their own environments [53]. Bendz [37] followed participants up to a year after stroke and found that participants did not want to give up hope: “*I'm going to train…feel that you mustn't give up…I do it to be able to…carry on existing…no good giving up…oh no.*” (page, 219). Similar expressions from Wottrich et al. [56] illustrated that the need for a concrete hope a patient has: “*My hope is to recover to my previous level, to be able to walk as well as before and to use the arm, to get as close as possible to where I was before, preferably all the way there. I'm not sure it's going to be possible, it's just training*” (page, 1220).

#### 3.2.5. Subtheme 2e: Success, Motivation, and Mastery Experiences

How participants perceived their success was dependent on what they had experienced moving forward and whether they were in a positive emotional state. The second order constructs of self belief and aspirations were enhanced when participants were achieving success [56]. Many studies reported participants experiencing a sense of momentum to recovery over time where the experience of some successful recovery helped to maintain their motivation [53–55]. Staff could be central to this, as Gibbon [51] illustrates “*My faith was in (named nurse)…he always made you get on and found ways to encourage you; he always lifted my spirits*” (page, 10).

#### 3.2.6. The Line of Argument Synthesis

**Figure 2:**
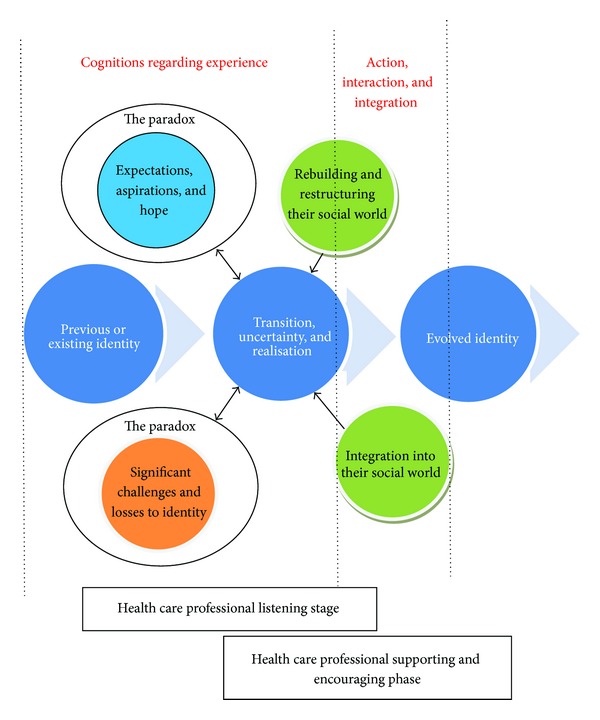
The model of transitional experiences within stroke rehabilitation.

Our synthesis of themes and subthemes clearly identifies a need for health care professionals to consider their interaction and care giving around the psychosocial needs of the patient in order to support their transition in identity. These needs are directly associated with the challenge the participant has in coming to terms with an altered identity.  Figure 2 provides details of the processes involved when the stroke influences a patient's identity.

The model of transitional experiences within stroke rehabilitation illustrates the transition in identity and factors which impact on that transition. Aligned with previous research in stroke and other neurological conditions [13] we utilise the paradox of chronic illness [65] to highlight that individuals are impelled to defy limitations imposed by their stroke but also concurrently have some understanding and acceptance of their limitations. Further to this, what an individual can accept about their situation and illness varies (inter-patient variation) and health care professional need to consider what a patient can accept.

Importantly for the health care professional, the model generated from this synthesis illustrates the need and value of considering the process of transition in identity and the importance of listening when there is uncertainty, expression of losses, or when an individual's expresses hope and aspirations. There is value in exploring such expressions to communicate to the patient that they have been heard and valued as an individual and, following this, attempting to change or help an individual that it is done in a style that uses questions to offer different or alternative views and possibilities (emphasis on negotiation with the patient) rather than confronting a patient and demanding change (emphasis on directing the patient). This is important because of the difficulty in or time needed when adapting to a new identity. Further, health care professions are vital in encouraging and supporting the individual when they attempt to integrate themself into their life following rehabilitation, as at these times, they may be most able to positively alter, adapt, and evolve their identity. Support and encouragement needs to reflect the use of psychosocial concepts which are identified within the respective theme.

## 4. Discussion

The current metaethnography has been able to consider how an individual's identity evolves and is influenced during stroke rehabilitation. We identified two themes which recognised that the individuals' identity is an essential aspect of perceived recovery and as such a central need that requires attention by clinicians and researchers alike. It is well known that individuals experience a process of reconstitution of self in response to the burden of living with chronic illness. Individuals actively engage by reflecting on their illness which helps them make sense of who they are, experiencing self in a newly conscious way [66–68]. Restoring a sense of control and self-sufficiency so an individual that feels able to rebuild and integrate their life is essential to moving on following a stroke. In this study several psychosocial constructs can be considered as factors which are influenced by the interaction with health care professionals. Within this discussion we focus on three specific constructs including social support, master experiences, and aspects that relate to self-efficacy and an individual's hope.

All the current studies except one [50] illustrated the importance of at least one dimension of social support. The four different dimensions [69] include emotional, esteem, information, and tangible; health care professionals may consider the need to match support to the situation a patient is in [69]. For instance, within uncontrollable situations for example, following diagnosis, emotional support may be best; however, once a patient perceives a more controllable situation, informational and tangible support may be best. Further the timing of support [70] illustrates different needs alongside different stressful situations [71] (Crisis, transition, and deficit (Crisis is defined as a situation that is severely threatening to ones well-being and incites emotional arousal, sudden onset, and limited duration. Transition is defined as a period of change in an individual's relationships or assumptive world. Deficit is a situation where one's life has chronically excessive demands.)). During crisis, emotional support can be provided, during transition, information support may be more appropriate, and during a deficit, tangible support could be provided.

Both instrumental and emotional support can influence the transitional model of stroke care model via improved autonomy. When external support is provided the transition to a reintegrated identity is more rapid and extensive [72]. Increased family support also results in patients being more informed and satisfied with the quantity of information and education provided [21, 73, 74]. Support positively impacts on autonomy and therefore self-esteem and well-being by preserving positive characteristics and attitude towards rehabilitation outcomes. In the absence of external support, which could be provided by recreation or employment, an individual may have a decreased sense of self-perception in conjunction with a decreased sense of belonging [26]. In order to experience the valuable sense of belonging high levels of self-efficacy are required to change behaviour and learn from social situations.

Key dimensions of an individual's self efficacy were identified within the current studies including the need for verbal persuasion in the form of encouragement and feedback from health care professionals [48, 51–54] and mastery experiences, in the form of a sense of momentum [26, 39, 49, 51, 53, 55, 56], increasing expertise [26, 37, 48, 49, 52, 54], and progress to goals [50, 51, 55, 56]. An individual's self-efficacy affects their ability to learn, improves their level of achievement, and determines their motivation [73, 75–77]. According to the social cognitive theory a high self-efficacy helps individuals to persevere, despite difficulties, to achieve the things they value which positively influences their health and well-being [75, 78]. The subdomains of self-efficacy require further consideration. (1) Mastery experiences are the most effective force in creating personal efficacy. Individuals with stroke require time to practice and reflect on performing functional abilities well [73, 76, 79]. Repeated failures can reduce self-efficacy. (2) Verbal encouragement and supportive comments to progress towards goals are essential. Although it often does not instil strong self-belief and can have a negative effect in a challenging environment [35] or can be affected by the sources credibility and expertise [76]. (3) Vicarious experience or modelling the behaviour of others can help induce a feeling that they can posses the capability to master comparable activities [35]. This highlights the need to consider the participant's experience within a rehabilitation setting. (4) Performing functional tasks creates physiological feedback for the participant. It is important that patients interpret such experience as positive, rather than negative. For instance walking more independently [80].

A number of studies in this synthesis included interpretations of data from participants discussing feelings of despondency or anxiety during the rehabilitation process [49, 50, 53–55]. This may be as a result of an individual having poor self-efficacy often associated with feeling a lack of control of one's situation. An inability to influence events may result in self-isolation and feelings of futility causing a decrease in confidence and an increasing restriction of social roles [81]. This may also be associated with the potential for a negative cycle when one's situation can be perceived as worsening.

Individuals in all studies recognised different psychological concepts and cognitions that directly related to hope. Factors that influenced hope included a focus on the progress towards goals [50, 51, 55, 56], the need to gain independence [49, 50, 52, 56], and finally in all studies except two [50, 52] the expectation in one's self. The model illustrates that patients' hopes are likely to be dependent on their previous identity and perceived losses. In accordance with the paradox of chronic illness [13], it is important to understand what an individual can accept about the loss they perceive and how it affects their identity. Further to this, it is important to recognise that in some instances the hope of a possible recovery may be an individual's main agency for motivation and challenging the effects of the stroke. Removing this hope for change can leave a patient with a very negative view of the therapist [82]. Rebuilding and restructuring one's identity relies on mastery of experiences, learning and self-development, where hope changes and adapts due to perceived success and being under the influence of others.

### 4.1. Clinical Implications

Several implications for health care professionals in clinical practice can be identified from the current results including a need to

1. be aware that an individual's previous identity will impact on how they adjust to their life with a stroke;
2. consider the importance of social support behaviours when a patient is adjusting to their stroke. Specifically understanding how to support an individual's confidence, autonomy towards rehabilitation activities, hope, and expectations;
3. understand the importance of self-efficacy subdomains;
4. understand that once established self-efficacy can generalise to other situations where these new perspectives can lead to changes in behaviour resulting in better overall health and function.

### 4.2. Limitations

All the synthesised studies had their own inclusion criteria for the focus groups or interviews and all primary data was collected from stroke survivors who did not suffer from any severe cognitive or language difficulties. This does bias the findings of the study in favour of survivors in the less severe stroke category decreasing the generalisability of the findings. Another limitation of this study was that the rehabilitation experience data was not relevant to a specific healthcare system or rehabilitation style, but in the majority reflected a European rehabilitation experience. In this review the findings have therefore been of a more general nature but this may reduce the study's usefulness for specific service development in a specific healthcare system. The line of argument synthesis was influenced by the supervising author's a priori analysis.

### 4.3. Clinical Implications

Patient experience is linked to quality of life measures such as well-being and is often dependent on an individual's sense of engagement with the healthcare system [23]. In this study positive experiences appear to be associated with an improved ability for participants to manage their condition and make positive health and well-being choices, highlighting the importance of subjective experiences. Rehabilitation occurs in the hospital setting, the home environment, and the local community where outcome measures form an essential part of the process both at the individual level of effective improvement and at service level in justification for continued input. In order to assess the effectiveness of rehabilitation on the subjective experiences highlighted in this model, increased emphasis on outcome measurement would be required.

## 5. Conclusion

The metaethnography synthesised and translated constructs from thirteen studies producing two themes and a model relating to the transitional experiences within rehabilitation. It is important to acknowledge the complexity and the multidimensional nature of the recovery process for stroke survivors. In this context designing interventions to facilitate recovery is not a simple matter. This model of transitional patient experience may be useful when considering the importance of valuing an individual. It may have educational value for professionals not used to prioritising patient experience and subjective beliefs in the rehabilitation process.

## Figures and Tables

**Table 1:** The database search terms.

Condition	Specific person	intervention	Result of intervention
Stroke	Patient*	Rehabilitation	Experience
CVA	Service user	Rehab*	Satisfaction
Cerebrovascular accident	Stroke survivor	Therapy	Well-being
	Client*	Physical therapy*	Perception
		Physiotherapy*	Contentedness
			Quality of therapy
			Quality of rehabilitation

Note: during the search each term within the column was combined using the term “OR” then the results from each column were combined using the term “AND.”

*A process in searches where the words were reduced in size to capture ali variants of the word. E.gv patient* could be patient, patients, patient's, patients'.

**Table 2:** Characteristics of the synthesised papers.

Source paper/location of study	Sample population (age and gender)	Data collection and schedule	Location of data collection	Specific topic covered in study	Method of analysis
(1) Sabari et al. (2000) [48] (USA)	Aged 45–75 years 6 stroke survivors; 5 male 4 female carers of stroke survivors	2 *Focus groups* (in place of regular support group meetings)	Home environment of one of the participants	Encouraged as normal as possible discussions as per support group meetings were found to be discussions about experiences of rehab	Grounded theory approach
(2) Proot et al. (2000) [49] (Netherlands)	17 stroke patients; aged 50–85 years; 7 female	*Interview* 6–11 weeks after admission to rehabilitation ward	Hospital setting	Open ended interview questions based on autonomy which patients were assisted to define for themselves initially How they defined autonomy Changes in time on persons autonomy Constraints and improvements to their autonomy Approach of health professionals and family to their autonomy	Grounded theory approach
(3) Burton (2000) [50] (U.K)	6 stroke patients; aged 52–81; 4 female	Monthly *interviews* for 12 months maximum post-stroke	Initially hospital then home environment	Informal and unstructured patient asked to tell their story	Grounded theory approach
(4) Bendz (2003) [37] (Sweden)	15 stroke patients All <65 years old; 9 male	*Interviews* 3, 6 and 12 months after admission from stroke	Home environment	Semi-structured questions re: patient's perception of their stroke and ensuing rehabilitation	Interpretative approach to create categories on analysis (grounded theory approach)
(5) Röding et al. (2003) [38] (Sweden)	5 stroke patients; aged 37–54; 3 male	*Interview *	Stipulated by participants: 4 in home environment	Hospital stay Rehabilitation period Current situation	Grounded theory approach
(6) Cowdell and Garrett, (2003) [39] (U.K)	8 stroke patients; age unknown	*Interview *	Hospital ward	Participant current levels of activity. Views on recreational activity prior to stroke and after-stroke	Grounded theory approach
(7) Gibbon, (2004) [51] (U.K)	15 stroke patients aged 47–84; 8 male	One *interview *	Home environment	Impact of stroke Meaning of rehabilitation Key contributors Goal setting Discharge home	Latent content analysis/grounded theory approach
(8) Olofsson et al. (2005) [52] (Sweden)	9 stroke patients; aged 64–83; 5 females	*Interviews* 1: 4 months after-stroke 2: following care at stroke centre	Home environment	Patient's stay in hospital Experiences of coming home Experiences of rehabilitation Experiences of follow-up appointments	Grounded theory approach
(9) Morris et al. (2007) [53] (U.K)	10 stroke patients; aged 45–81; 8 male 5 carers; aged 45–83; 3 female	A *focus group *(1.5 hours)	Hospital setting	Chronological questions sequenced from the stroke event to discharge	Grounded theory approach
(10) Mangset et al. (2008) [54] (Norway)	12 stroke patients; aged 60–87 yrs; 7 female	*Interviews* (1–6 weeks after admission; 3 months later)	Initially hospital setting followed by home environment	Share experiences in connection with the stroke incident Experiences of being a stroke patient Experience of the health service Experience of the rehabilitation process	Grounded theory approach
(11) Ellis-Hill et al. (2009) [55] (U.K)	20 stroke patients 13 carers	*Interview* within a month of discharge	Home environment	Talk about the effects of their stroke Their priorities for recovery Their views about therapy and services	Framework analysis with the grounded theory approach
(12) Erikson et al. (2010) [26] (Sweden)	9 stroke survivors; Aged 42–61 years; 6 male	*Interviews* (1, 3, 6 and 12 months)	At 1 month: hospital setting At 3, 6, and 12 months: outpatient clinic	Experiences performing daily activities relative to those experiences prior to acquiring a stroke Experiences of daily life with stroke	Grounded theory approach
(13) Wottrich et al. (2012) [56] (Sweden)	5 stroke patients; aged 44–70 years; 3 female	*Interviews* 1: 1–7 days prior to discharge 2: 13–30 days after discharge 3: 3–4 months after discharge	Interview 1: hospital setting Interviews 2 and 3: home environment	Experience of ending contact with staff on the ward Experience of being discharged and coming home and of being at home Experience of everyday life today Strategies for handling a changed everyday situation/life situation Conceptions about one's future everyday situation/life situation Experience of important aspects that help or hinder progress in adapting to a changed everyday situation/life situation	Grounded theory approach

**Table 3:** The summary of results of the COREQ (Tong et al., 2007 [62]) appraisal for the thirteen included studies.

Author/year of publication	Domain 1 (8) Research team and reflexivity	Domain 2 (15) Study design	Domain 3 (9) Analysis and findings	Total (32)
Sabari et al. (2000) [48]	6	13	8	27
Proot et al. (2000) [49]	3	11	8	22
Burton (2000) [50]	4	12	6	22
Bendz (2003) [37]	5	10	7	22
Röding et al. (2003) [38]	3	10	8	21
Cowdell and Garrett (2003) [39]	6	11	6	23
Gibbon (2004) [51]	5	11	6	22
Olofsson et al. (2005) [52]	4	12	6	22
Morris et al. (2007) [53]	6	11	8	25
Mangset et al. (2008) [54]	3	12	6	21
Ellis-Hill et al. (2009) [55]	6	12	7	25
Erikson et al. (2010) [26]	6	12	6	24
Wottrich et al. (2012) [56]	5	10	7	22

Mean	**4.8**	**11.3**	**6.9**	**22.9**
Median	**5**	**11**	**7**	**22**
Mode	**6**	**12**	**6**	**22**

**Table 4:** Findings of the synthesised translations.

Theme	Subtheme	Second order construct	Papers where second order construct appears
(1) Evolution of identity	(a) Sense of the individual's identity	The whole person Individual factors for example, co-morbidities Normal activities and “old” life Individual recovery model	1, 2, 8, 9, 10, 12 2, 5 2, 3, 4, 11, 12, 13 1, 4, 6, 7, 10, 11, 13
	(b) Being at a dividing line	Transition Uncertainty Self realisation	1, 10, 12, 13 3, 4, 8, 11, 13 2, 5, 10, 11, 12, 13
	(c) Rebuilding and restructuring identity	Adjustment Value in experience Desire for re-integration Adaptation and practice Insight	2, 3, 5, 10, 12, 13 7, 12 7, 8, 11, 12 2, 3, 7, 11, 12, 13 5, 8, 10
	(d) Reintegrating identity with their life	Occupation Sense of identity Sense of belonging	12, 13 4, 11, 12, 13 5, 12

(2) Psychosocial constructs that influence experience	(a) Support	Information and resources Timely and long term support Emotional and instrumental support Network of people Feedback and encouragement	1, 4, 5, 6, 7, 8, 9, 10, 11, 13 1, 8, 11, 12 2, 8, 11, 12, 13 1, 2, 5, 8, 11, 12 1, 7, 8, 9, 10
	(b) Autonomy	Problem solving Self-confidence, determination, and self-esteem Shared decision making Partnership relationships Choice Focus on quality of life and recreation	2, 8, 11 2, 7, 8, 12, 13 2, 10, 11 2, 9, 10, 11, 12 1, 4, 12, 13 1, 6, 12
	(c) Coping and adjustment	Being another persons Loss of role and belonging Loss of control The paralysed everyday Emotions; “burden of burden,” helplessness Isolation	3, 4, 5, 8, 12 3, 6, 12 6, 8 3, 5, 6, 12 3, 5, 7, 8, 10, 12 4, 8, 12
	(d) Hope	Progress to goals Expectations (self, staff and treatment) Dependence to independence Continued stimulation High treatment priority	3, 7, 11, 13 1, 2, 4, 5, 6, 7, 9, 10, 11, 12, 13 2, 3, 8, 13 2, 3, 6, 9, 13 1, 2, 5, 9, 10, 13
	(e) Success motivation and mastery experiences	Increasing expertise Self-belief Motivation Sense of momentum Aspirations	1, 2, 4, 8, 10, 12 2, 10, 11, 13 2, 3, 4, 10 2, 6, 7, 9, 11, 12, 13 2, 7, 13

Note: 1: Sabari et al., 2000 [48], 2: Proot et al., 2000 [49], 3: Burton, 2000 [50], 4: Bendz, 2003 [37], 5: Röding et al., 2003 [38], 6: Cowdell and Garrett, 2003 [39], 7: Gibbon, 2004 [51], 8: Olofsson et al., 2005 [52], 9: Morris et al., 2007 [53], 10: Mangset et al., 2008 [54], 11: Ellis-Hill et al., 2009 [55], 12: Erikson et al., 2010 [26], and 13: Wottrich et al., 2012 [56].
